# Enhanced Bio-Ethanol Production from Industrial Potato Waste by Statistical Medium Optimization

**DOI:** 10.3390/ijms161024490

**Published:** 2015-10-15

**Authors:** Gulten Izmirlioglu, Ali Demirci

**Affiliations:** 1 Department of Agricultural and Biological Engineering, the Pennsylvania State University, University Park, PA 16802, USA; E-Mail: gxi111@psu.edu; 2 The Huck Institutes of Life Sciences, the Pennsylvania State University, University Park, PA 16802, USA

**Keywords:** bio-ethanol, *Saccharomyces cerevisiae*, industrial waste, Plackett-Burman, Box-Behnken design

## Abstract

Industrial wastes are of great interest as a substrate in production of value-added products to reduce cost, while managing the waste economically and environmentally. Bio-ethanol production from industrial wastes has gained attention because of its abundance, availability, and rich carbon and nitrogen content. In this study, industrial potato waste was used as a carbon source and a medium was optimized for ethanol production by using statistical designs. The effect of various medium components on ethanol production was evaluated. Yeast extract, malt extract, and MgSO_4_·7H_2_O showed significantly positive effects, whereas KH_2_PO_4_ and CaCl_2_·2H_2_O had a significantly negative effect (*p*-value < 0.05). Using response surface methodology, a medium consisting of 40.4 g/L (dry basis) industrial waste potato, 50 g/L malt extract, and 4.84 g/L MgSO_4_·7H_2_O was found optimal and yielded 24.6 g/L ethanol at 30 °C, 150 rpm, and 48 h of fermentation. In conclusion, this study demonstrated that industrial potato waste can be used effectively to enhance bioethanol production.

## 1. Introduction

Alternative fuels are of great interest due to the price fluctuations of petroleum-based fuels, government regulations on carbon dioxide emissions, and future depletion of petroleum reserves. 

Biofuels such as ethanol, methanol, and biodiesel are considered as alternatives to petroleum fuels, and worldwide production of fuel ethanol reached 91 billion liters in 2013 [1]. Current ethanol production depends on first generation crops, such as sugar cane, corn, wheat, cassava, and is commercialized globally with approximately 650 plants with a total capacity of 100 billion liters [2]. Corn-based ethanol represents the major fraction of ethanol production with 60 billion liters, followed by sugar cane-based ethanol with 20 billion liters in 2012 [2]. Uses of the first generation crops for ethanol production raise concerns over limited agricultural land and water, as well as other environmental issues in regards to land use [2,3]. Although second and third generation feedstocks, lignocellulosic biomass, and algae, respectively, have been considered as alternatives to the first generation crops, ethanol production from these feedstocks is not cost-competitive yet. Therefore, the agro-industrial wastes for ethanol production have been considered as carbon source.

Agro-industrial wastes have drawn attention for ethanol production due to their abundance, availability, biodegradability, rich carbon and nutrient content, and also to manage the waste issues of industry economically and environmentally [4]. Waste and by-products of the potato industry have potential for fermentation industry due to their high starch content and availability. In the potato processing industry, 50% of the potatoes are generally wasted [5]. However, the percentage varies for different potato processing plants, e.g., potato processing industry creates 10% of waste potato pulp [6], 5%–20% cull potatoes [7], and 15%–40% peel [8]. In the potato chips industry, on the other hand, 18% of the production is starchy waste [9]. The use of industrial potato waste (including potato peels, potato mash, potato pulp, and potato processing wastewater) for the production of α-amylase, lactic acid, and pullulan have been reported [8,9,10,11].

The production of ethanol from several agro-industrial wastes has been reported. These include food waste [12,13], banana peels [14], carob extract [15], pineapple waste [16], potato peel [8], and waste of the olive oil industry [17]. Moreover, ethanol production from potatoes and potato peels has been investigated. Rani *et al.* [18] have studied ethanol fermentation from potato flour and reported 56.8 g/L ethanol production from liquefied slurry (250 g/L potato flour) at 30 °C for 48 h by *S. cerevisiae* HAU-1 without nitrogen supplementation. Another study was undertaken for utilization of potato peel waste, and 7.6 g/L ethanol was produced after enzymatic hydrolysis of potato peels [8]. Izmirlioglu and Demirci [19] have studied the ethanol production from waste potato mash, and saw that it produced a maximum of 30.99 g/L ethanol with enzymatically hydrolyzed waste potato mash at pH 5.5 and 30 °C after 48 h of fermentation. These conditions reveal 0.44 g ethanol/g glucose, which is equal 86.7% of the theoretical yield. Because medium cost represents a major fraction of fermentation costs, medium optimization for ethanol from industrial waste potato still needs to be investigated, since there has been no study that has an optimized medium for ethanol from industrial waste potato mash by *Saccharomyces cerevisiae* in particular.

Medium optimization by the conventional methods, which usually studies one variable at a time is not only time-consuming, but also laborious, and impractical. Statistical designs are commonly utilized for medium optimization studies and are considered a better way of interpreting the results than traditional one-variable-at-a-time studies. Statistical designs can also be applied at the different phases of the optimization process, such as during the screening of a large number of variables in the medium, or optimization of the growth parameters such as temperature, pH, and agitation to achieve the maximum production. For screening of a large number of variables, the Plackett-Burman statistical design [20] is well-known and commonly employed by researchers. Reliable information in regards to investigate the linear effects of the variables can be obtained with the Plackett-Burman design and reduced number of medium components can be further optimized. On the other hand, Box-Behnken design [21] using response surface methodology can be employed for further optimization of selected medium components, while considering the linear and quadratic interactions among the variables.

This study, therefore, investigates industrial waste potato mash as the carbon source, and the medium optimization for ethanol production. To conduct this study, the Plackett-Burman statistical design was employed to screen for the effects of various medium ingredients on ethanol production and further optimization of the medium was conducted using response surface methodology, the Box-Behnken design.

## 2. Results

### 2.1. Effects of Medium Components on Ethanol Production

**Figure 1:**
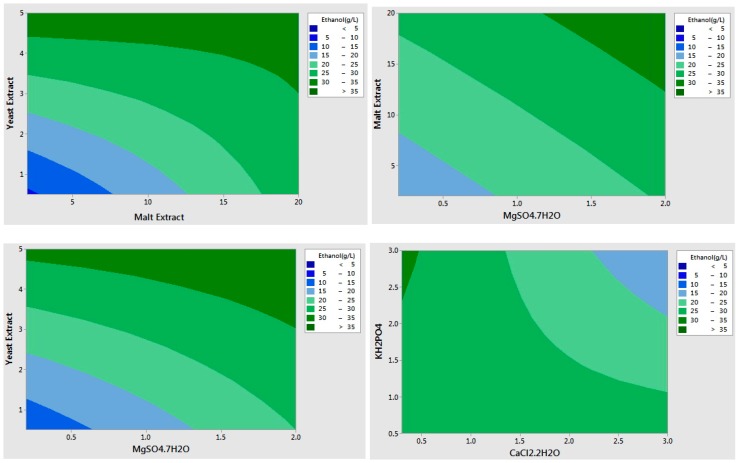
Plackett-Burman counter plots showing individual effects of statistically-significant factors on bio-ethanol production.

Eight medium components that are chosen from the literature were examined using the Plackett-Burman statistical design (Table 1). The experimental design matrix for screening of important medium components and the experimental results are shown in Table 2. The ethanol production by *S. cerevisiae* varied from 5.05 to 36.85 g/L in 12 experiments, which proves the importance of medium components and their concentrations on ethanol production. The analysis showed that medium #3 resulted in the highest ethanol production (36.85 g/L), followed by medium #6 and medium #1 (36.8 and 31.5 g/L, respectively). Either malt extract or yeast extract were at high levels (20 and 5 g/L) in all of these medium compositions, showing the impact of those components. Media #2, #7, and #12 demonstrated the lowest ethanol production (5.05, 8.36, and 14.15 g/L, respectively). All of the three media consisted of low concentration of yeast extract, which demonstrates its significance. The main effects, regression coefficients, *f*-values, and *p*-values of each variable are given in Table 3. According to these Analysis of variance (ANOVA) results, the total of five variables—yeast extract, malt extract, MgSO_4_·7H_2_O, KH_2_PO_4_, and CaCl_2_·2H_2_O—showed statistically significant effects on ethanol production (*p*-value < 0.05), while effects of (NH_4_)_2_SO_4_, CaCO_3_, and FeSO_4_·7H_2_O were not statistically significant. The analysis of coefficients showed that yeast extract, malt extract, and MgSO_4_·7H_2_O had positive effects on ethanol fermentation, while KH_2_PO_4,_ and CaCl_2_·2H_2_O exerted negative effects (*p*-value < 0.05). The most significant variable was yeast extract, which had the highest coefficient, followed by malt extract and MgSO_4_·7H_2_O (Table 3). Figure 1 shows the counter plots of the effects of the various combinations of the statistically-significant independent variables on ethanol production by *S. cerevisiae*. These plots also clearly demonstrated the positive and the negative effects of the statistically-significant factors. Therefore, the ingredients with no significant effect and the ingredients with significantly negative effects on ethanol production were left out from the fermentation medium, and the concentrations of yeast extract, malt extract, and MgSO_4_·7H_2_O were further optimized by Surface Response Methodology. Please note that industrial waste potato mash was used as a carbon source instead of glucose for the rest of the study.

**Table 1:** Concentrations of variables at high and low levels in Plackett-Burman design *.

Variable	Lower Level	High Level	Reference
Yeast Extract (g/L)	0.5	5	[22]
Malt Extract (g/L)	2	20	[23]
(NH_4_)_2_SO_4_ (g/L)	2	6	[8]
MgSO_4_·7H_2_O (g/L)	0.2	2	[24]
KH_2_PO_4_ (g/L)	0.5	3	[8]
CaCO_3_ (g/L)	0.2	2	[25]
FeSO_4_·7H_2_O (g/L)	0.01	0.1	[26]
CaCl_2_·2H_2_O (g/L)	0.3	3	[26]

* The medium also includes 100 g/L glucose as the carbon source.

**Table 2:** Placket-Burman experimental design matrix for screening of important variables for bio-ethanol production with results *.

Medium Number	Yeast Extract (g/L)	Malt Extract (g/L)	MgSO_4_·7H_2_O (g/L)	(NH_4_)_2_SO_4_ (g/L)	KH_2_PO_4_ (g/L)	CaCO_3_ (g/L)	FeSO_4_·7H_2_O (g/L)	CaCl_2_·2H_2_O (g/L)	Ethanol (g/L)
1	5	20	2	2	3	2	0.01	3	33.5
2	0.5	2	0.2	6	3	2	0.01	3	5.05
3	0.5	20	2	6	0.5	2	0.1	0.3	36.85
4	0.5	20	0.2	2	0.5	2	0.1	3	15.9
5	5	2	2	6	0.5	2	0.01	0.3	31.45
6	5	2	2	2	0.5	0.2	0.1	3	36.8
7	0.5	2	2	6	3	0.2	0.1	3	8.36
8	5	20	0.2	6	3	0.2	0.1	0.3	31.95
9	5	20	0.2	6	0.5	0.2	0.01	3	30.45
10	0.5	20	2	2	3	0.2	0.01	0.3	29.8
11	5	2	0.2	2	3	2	0.1	0.3	31.2
12	0.5	2	0.2	2	0.5	0.2	0.01	0.3	14.15

* The medium also includes 100 g/L glucose as the carbon source.

**Table 3:** Statistical analysis of Plackett-Burman design for ethanol production from industrial waste potato mash by *S. cerevisiae.*

Variables	Main Effect	β-Coefficients	*f*-Value	*p*-Value
Yeast Extract (g/L)	14.206	7.103	71.87	0.000
Malt Extract (g/L)	8.573	4.286	26.17	0.000
(NH_4_)_2_SO_4_ (g/L)	−2.873	−1.436	2.94	0.109
MgSO_4_·7H_2_O (g/L)	8.009	4.004	22.84	0.000
KH_2_PO_4_ (g/L)	−4.289	−2.144	6.55	0.023
CaCO_3_ (g/L)	0.407	0.203	0.06	0.811
FeSO_4_·7H_2_O (g/L)	2.776	1.388	2.74	0.120
CaCl_2_·2H_2_O (g/L)	−7.556	−3.778	20.33	0.000

### 2.2. Optimization of the Selected Medium Components Using Response Surface Methodology

Further optimization of ethanol fermentation by *S. cerevisiae* was carried out in a waste potato mash of hydrolysate-based medium after screening of the medium components by the Plackett-Burman design. Based on the results of the Plackett-Burman design, yeast extract, malt extract, and MgSO_4_·7H_2_O were optimized using Response Surface Methodology’s Box-Behnken design. The experimental matrix and the results of Box-Behnken experiments are presented in Table 4. The highest ethanol production (32.52 g/L) was observed when waste potato mash hydrolysate was supplemented with 50 g/L malt extract, 5 g/L MgSO_4_·7H_2_O, and 0 g/L yeast extract. In contrast, the medium composition which consisted of 25 g/L yeast extract, 5 g/L MgSO_4_·7H_2_O, and 0 g/L malt extract resulted in the lowest ethanol production (12.93 g/L). Malt extract showed a strong influence on ethanol production. Ethanol concentrations varied from 12.93 to 26.21 g/L depending on the concentration of malt extract, specifically when yeast extract and MgSO_4_·7H_2_O were kept constant at 25 and 5 g/L, respectively. Higher malt extract (50 g/L) enhanced ethanol production and resulted in 26.21 g/L ethanol, whereas ethanol production dropped to 12.93 g/L in the absence of malt extract. The same pattern was observed for MgSO_4_·7H_2_O, and 22.95 g/L ethanol was produced when the medium supplemented with 10 g/L MgSO_4_·7H_2_O, 25 g/L malt extract, and 25 g/L yeast extract. However, 18.85 g/L ethanol was produced when MgSO_4_·7H_2_O was not added to the medium even though concentrations of yeast and malt extracts were the same. The data showed that yeast extract did not have any significant impact on ethanol production in the evaluated range when waste potato mash hydrolysate was used as a carbon source. When malt extract and MgSO_4_·7H_2_O concentrations were kept constant at 50 and 5 g/L, respectively, 32.52 g/L ethanol was obtained in the absence of yeast extract. Ethanol concentration, however, decreased to 26.21 g/L when 25 g/L yeast extract was added to the medium. Even though ethanol production was highly influenced by medium compositions in the design matrix, cell population did not show a significant difference among runs (Table 4).

**Table 4:** Box-Behnken experimental design matrix with the experimental values of bio-ethanol production *.

Run Order	Yeast Extract (g/L)	Malt Extract (g/L)	MgSO_4_·7H_2_O (g/L)	Ethanol (g/L)	Cell Population (log CFU/mL)
1	25	25	10	22.95	7.14
2	12.5	50	0	26.01	7.15
3	0	0	5	13.77	6.02
4	12.5	25	5	22.37	7.19
5	25	50	5	26.21	7.13
6	12.5	25	5	24.71	7.26
7	0	25	0	18.83	6.82
8	25	25	0	18.85	7.00
9	0	25	10	20.50	7.16
10	12.5	25	5	28.59	5.95
11	12.5	0	0	15.41	6.10
12	25	0	5	12.93	6.02
13	12.5	50	10	20.57	7.05
14	12.5	0	10	13.86	6.19
15	0	50	5	32.52	7.07

* The medium also includes 40.4 g/L (d.b.) industrial potato mash as the carbon source.

**Figure 2:**
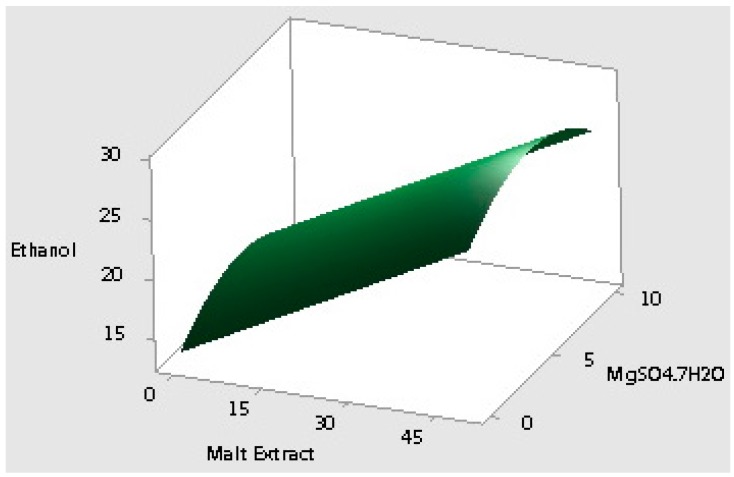

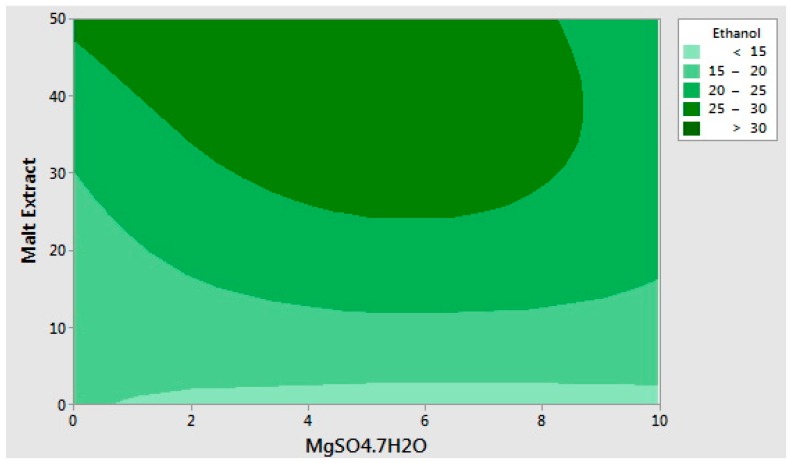
Response surface and contour plots for ethanol production showing the interaction of malt extract and MgSO_4_·7H_2_O concentrations and their effects on the bio-ethanol production.

**Figure 3:**
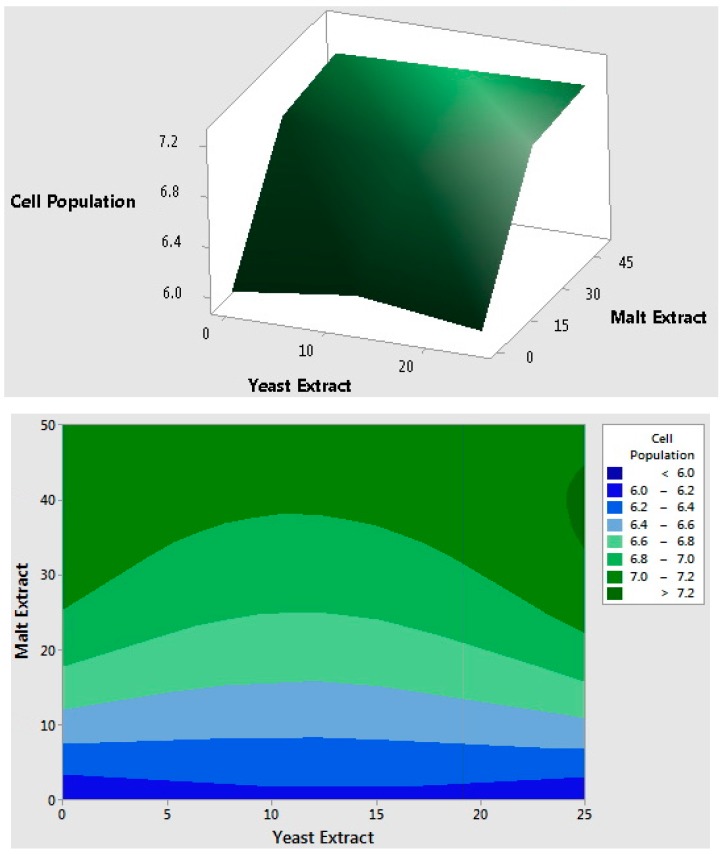
Response surface and contour plots for cell population showing the interaction of malt extract and yeast extract concentrations and their effects on the cell population (MgSO_4_·7H_2_O at mid-value).

The regression analysis of the ethanol production and cell population data were represented by a second order polynomial equation without the insignificant terms as shown below:

(1) $$Ethanol\;\left(\frac{g}{L}\right)=13.61+0.24\;X_{2}+X_{3}-X_{3}^{2}$$

(2) $$Cell\;Population\;\left(\frac{CFU}{L}\right)=6.082+0.0476\;X_{2}-X_{2}^{2}$$

where *X*_2_ and *X*_3_ are malt extract (g/L) and MgSO_4_·7H_2_O (g/L), respectively. The results of ANOVA for ethanol production indicated that the model is reliable with a 73.8% *r*-square and a 0.002 *p*-value with significant linear and quadratic effects when insignificant effects are excluded. Statistical analysis showed that ethanol production was highly affected by malt extract and MgSO_4_·7H_2_O, but the effect of yeast extract was not significant (*p-*value > 0.1). Linear coefficient of malt extract was highly significant (*p-*value < 0.000). In addition, ethanol production demonstrated a non-linear effect with the increase of MgSO_4_·7H_2_O from 0 to 10 g/L under the studied conditions. The ethanol production reached a peak value (>30 g/L) at the mid-value of MgSO_4_·7H_2_O (5 g/L). Response surface and counter plots are graphical representations of the regression model equation for ethanol production (Figure 2), and linear effect of malt extract and quadratic effect of MgSO_4_·7H_2_O can be seen clearly. The ANOVA results of cell population data, on the other hand, showed that only malt extract had a significant effect on cell population (*p*-value < 0.05), while yeast extract and MgSO_4_·7H_2_O had no statistical significance (*p*-value > 0.05). The regression model of cell population had an *r*-square value of 66.2%. Malt extract showed a linear effect on cell population; however, increasing the malt extract concentration above 25 g/L did not increase the cell population further (Figure 3).

**Figure 4:**
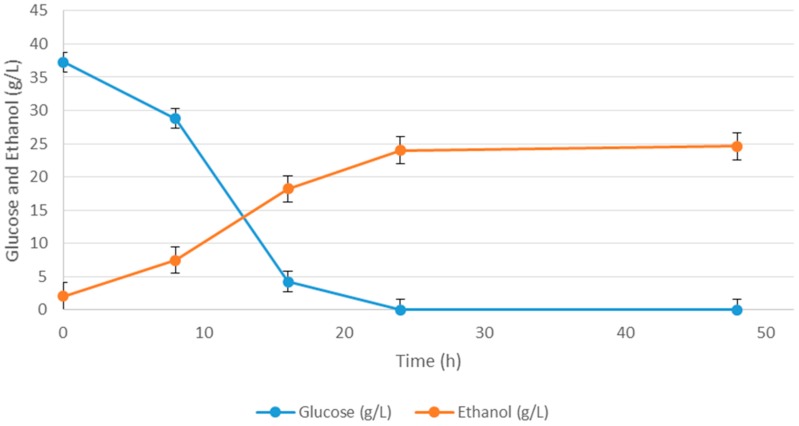
Bio-ethanol production and glucose consumption using the statistically optimized medium.

The response optimizer tool in Minitab was used to determine the optimal medium composition to maximize the production of ethanol. The optimum conditions were found to be 50 g/L of malt extract and 4.84 g/L of MgSO_4_·7H_2_O with no yeast extract, which yielded 24.6 g/L ethanol. Because the optimum amount of malt extract (50 g/L) found to be at the high end of the tested range (0–50 g/L), another set of fermentation performed to determine whether further increase in malt extract levels would improve the ethanol yield or not. To conduct this study, only malt extract concentrations increased up to 100 g/L (50, 60, 70, 80, 90, 100 g/L) in the optimal medium. No further improvement in ethanol yields was observed, but there was even a slight decrease in ethanol yield (data not shown). Therefore, it was concluded that the optimum concentration of malt extract is 50 g/L. The experimental time course of optimal medium composition is presented in Figure 4. Because glucose was used as a carbon source for the Plackett-Burman design experiments, a comparison study was conducted where the waste potato mash hydrolysate was used as the sole carbon source. Enhancement of ethanol production with the medium optimization can be seen in Table 5, where ethanol production was increased from 11.63 to 24.6 g/L. 

**Table 5:** Comparison between the basal and optimized media.

Media	Ingredient Name	Ingredient Concentration (g/L)	Ethanol (g/L)
Basal	Waste potato mash	40.4 (dry weight)	11.63
Basal Plackett-Burman validation media	Waste potato mash	40.4 (dry weight)	17.03
	Yeast Extract	5	
	Malt Extract	20	
	MgSO_4_·7H_2_O	2	
Response Surface validation media	Waste potato mash	40.4 (dry weight)	24.6
	Yeast Extract	0	
	Malt Extract	50	
	MgSO_4_·7H_2_O	4.84	

## 3. Discussion

The potato is an organic compound and composed of carbohydrates, proteins, fat, carotene, thiamine, riboflavin, and ascorbic acid [27], and these components might promote the cell growth and product formation during ethanol fermentation (Table 5). In order to determine the effect of medium ingredients, glucose was used as the carbon source instead of the potato waste mash hydrolysate during the Plackett-Burman screening. This way, the influence of the medium ingredients could be observed without interactions of nutrients that would have otherwise leached from the waste potato mash. 

Nitrogen is one of the key nutrients in ethanol fermentation by *S. cerevisiae* for protein synthesis and cell growth [22,23]. Nitrogen, furthermore, affects the alcohol tolerance of the yeast, as well as the production rate [28]. *S. cerevisiae* has developed a wide range of nitrogen regulation systems and can use up to 30 different nitrogen containing compounds [29,30]. The source of nitrogen, however, is important in terms of by-product formation: glycerol. Ammonium salts, when used as the sole nitrogen source, may increase the glycerol formation whereas amino acids tend to decrease [31]. This might explain the negative effect of ammonium sulfate on ethanol production during the Plackett-Burman screening experiments. Pereira *et al.* [26] and other researchers [32] observed a similar pattern when studying medium optimization for very high gravity ethanol fermentation and reported that ammonium sulfate decreased ethanol concentration. In contrast, yeast extract showed statistically significant positive effects. Yeast extract is well known for its high nitrogen content (>75%) [23], and has been studied extensively. The effect of media supplementation for very high gravity ethanol production by *S. cerevisiae* was studied by Bafrncová *et al.* [28]. The authors reported that a 17% increase in ethanol production was observed when the concentration of free amino nitrogen increased. Also, higher glucose consumption rate and biomass yield was observed with an increase in yeast extract concentration (from 3 to 9 g/L). Thomas and Ingledew [33] studied very high gravity fermentation of wheat mash and reported a 21% ethanol yield in four days when the medium was supplemented with 1% yeast extract. In another study [34], the highest ethanol concentration (36 g/L) was achieved in the presence of 10 g/L yeast extract when food waste was utilized as feedstock. A decrease in the ethanol concentration was also observed when the yeast extract concentration was decreased to 2.5 g/L, in the same study [34]. These studies align with our Plackett-Burman screening results, and show the importance of nitrogen source for the ethanol production.

On the other hand, the concentration of nitrogen source is another important factor for efficient ethanol fermentation. Although yeast extract promoted ethanol fermentation when glucose was used as sole carbon source, addition of yeast extract was found unnecessary when waste potato mash was used as a carbon source during response surface optimization. The nitrogen content of potato tubers has been reported as 4.5% [35]. This might be due to sufficient nitrogen levels of industrial waste potato mash in combination with the malt extract. Rani *et al.* [18] reported that no nitrogen supplementation was necessary for ethanol fermentation from potato flour, which is in agreement with our results. Vilanova *et al.* [36] also reported that excessive amounts of nitrogen decreased the ethanol concentration. Furthermore, high concentrations of yeast extract did not promote ethanol production when nitrogen supplementation for very high gravity ethanol production was studied [23].

Malt extract showed a significantly positive effect in the Plackett-Burman screening test, as well as in Response Surface Box-Behnken experiments. The positive effects of malt extract may be due to its sugar and nitrogen contents that promote biomass growth and result in higher ethanol yields. Nitrogen content of malt extract may vary from 1.4%–1.8% according to O’Rourke [37]. This amount may be insufficient itself, but when combined with the nitrogen of waste potato mash, it was found to be satisfactory for *S. cerevisiae* for both ethanol production and cell population. Further increases in malt extract (up to 100 g/L), caused a slight decrease in ethanol production which can be explained that high levels of nitrogen might decrease ethanol yields as reported in other publications [36].

Magnesium sulfate also showed a significant effect on ethanol fermentation not only in the Plackett-Burman screening, but also in Box-Behnken optimization, which is in agreement with several publications that reported the positive effect of magnesium ions on ethanol production. It is reported that magnesium ions prolonged exponential growth and promoted yeast cell mass and enhance fermentative activity during batch cultures [24]. Pereira *et al.* also reported that MgSO_4_·7H_2_O increased the final ethanol concentrations; however, increased concentrations of MgSO_4_·7H_2_O resulted in a decrease in ethanol production. Authors reported 3.8 g/L of MgSO_4_·7H_2_O was optimum for very high gravity ethanol fermentation by *S. cerevisiae* [26].

In this study, it was demonstrated that 11.63 g/L ethanol can be produced from 40.4 g/L (d.b.) industrial waste potato mash without any supplementation after an enzyeme hydrolysis. However, supplemention of a medium with 50 g/L malt extract and 4.84 g/L MgSO_4_·7H_2_O increased the ethanol concentration and resulted in 24.6 g/L ethanol at the end of 48 h fermentation. Our results show that the screening and optimization methodologies described here enhanced the ethanol production from inudstrial waste potato mash by *S. cerevisiaie* without yielding higher biomass. Further studies may be conducted to substitue the malt extract with an inexpensive source of nitrogen, as well as increased solid loading.

## 4. Experimental Section

### 4.1. Microorganisms and Inoculum Preparation

The yeast, *Saccharomyces cerevisiae* (ATCC 24859) was purchased from the American Type Culture Collection (Manassas, VA, USA). Inoculum preparation was carried out as follows: *S. cerevisiae* was grown in a medium including 20 g/L of glucose, 6 g/L of yeast extract (Difco, Sparks, MD, USA), 0.3 g/L of CaCl_2_·2H_2_O, 4 g/L of (NH_4_)_2_SO_4_, 1 g/L of MgSO_4_·7H_2_O, and 1.5 g/L of KH_2_PO_4_ at 30 °C for 24 h. Working culture was maintained by storing at 4 °C and sub-cultured every two weeks, while stock cultures were stored in 20% glycerol at −80 °C.

### 4.2. Industrial Waste Potato Mash

A local potato processing plant provided the waste potato mash, which was utilized as the carbon source in the medium. Mash potato wastes were from different potato varieties, such as Frito-Lay FL 1833, Snowden, and Russet Burbank potatoes. The moisture analysis showed that the waste potato mash consisted of 17%–24% of starch. No pretreatment was carried out prior to fermentation, and the waste potato mash stored at −20 °C until its use.

### 4.3. Hydrolysis of Starch

α-Amylase (EC 3.2.1.1) and amyloglucosidase (EC 3.2.1.3) were employed for liquefaction and saccharification, respectively, during the hydrolysis of starch. These enzymes were kindly provided by Pennsylvania Grain Processing, LLC^®^ (Clearfield, PA, USA). Enzymatic hydrolysis was performed in two steps [19]. Industrial waste potato mash slurry was prepared (40.4 g/L (d.b.)), and alpha amylase was added (590 U/g dry substrate) to the slurry for liquefaction. Liquefaction was carried out at 95 °C for 3 h in an autoclave. In the second step, saccharification was carried out with the addition of amyloglucosidase (25 U/g dry substrate) after the liquefied slurry cooled down. Saccharification was carried out at 60 °C, 150 rpm for 72 h in a shaker incubator (SHKE5000-7, Barnstead International, Dubuque, IA, USA).

### 4.4. Experimental Design

#### 4.4.1. Plackett-Burman Design

The Plackett-Burman statistical design was used in this study as a first step of medium optimization to determine the statistically significant medium constituents on ethanol production. For screening of medium components, eight variables—malt extract, yeast extract, MgSO_4_·7H_2_O, (NH4)_2_SO_4_, KH_2_PO_4_, CaCO_3_, FeSO_4_·7H_2_O, and CaCl_2_·2H_2_O—were selected. Those medium ingredients were used to supplement the medium for ethanol fermentation by *S. cerevisiae* in the literature (Table 1). Because potato waste is an organic complex source and contained some nutrients, in this part of study, glucose (100 g/L) was used as a carbon source to determine the sole effect of each ingredient. This way, the authors ensured that the observed effect of each ingredient did not interact with nutrients that may be leached from potato waste.

The Plackett-Burman design allows studying N-1 factors with N experiments. In the design, each factor is represented at the high (+) level and the low (−) level. The calculation of the effect of each variable is shown in Equation (3),

(3) $$E=\frac{\sum^{\text{​}}y\left(+\right)}{N}-\frac{\sum^{\text{​}}y\left(-\right)}{N}$$

where *E* is the effect of the factor, *y*(*+*) and *y*(−) are the responses when a given factor is at its high and low levels, respectively, and *N* is the total number of experiments. As illustrated in Equation (1), the Plackett-Burman design is only for screening purpose due to the fact that the interactions among the factors are neglected.

Two levels (low and high) for each factor were studied (Table 1). Also, 12 medium compositions suggested by the Plackett-Burman design, were investigated (Table 2). Fermentation was carried out in 250 mL flasks (with a 100-mL working volume) at 30 °C, 150 rpm for 48 h in a shaker incubator (Barnstead International). Each experiment was replicated in triplicate. Minitab Statistical Software (Version 16.1; Minitab Inc., State College, PA, USA) was employed for statistical analysis. After analysis of variance (ANOVA), statistically significant effects were identified and validated. 

#### 4.4.2. Response Surface Methodology

Medium ingredients (yeast extract, malt extract, and MgSO_4_·7H_2_O) affecting bio-ethanol production, as suggested by the Plackett-Burman design, were further optimized using the Box-Behnken design of response surface methodology via Minitab (Version 16.1). The levels of these factors are given in Table 3. The independent variables were coded as *X*_1_ (yeast extract), *X*_2_ (malt extract), and *X*_3_ (MgSO_4_·7H_2_O), and the second-order model was used to predict the response to the independent variables (Equation (4)).

(4) $$y={\text{β}}_{\begin{array}{c} 0 \end{array}}+{\text{β}}_{1}X_{1}+{\text{β}}_{2}X_{2}+{\text{β}}_{3}X_{3}+{\text{β}}_{11}X_{1}^{2}+{\text{β}}_{22}X_{2}^{2}+{\text{β}}_{33}X_{2}^{2}+{\text{β}}_{12}X_{1}X_{2}+{\text{β}}_{13}X_{1}X_{3}+{\text{β}}_{23}X_{2}X_{3}$$

where *y* is the response (ethanol), β_0_, β_i_, and β_ii_ are the regression coefficients.

Fermentation runs were carried out in 250 mL flasks with a working volume of 100 mL. Industrial waste potato mash was hydrolyzed as described and supplemented with malt extract, yeast extract, and MgSO_4_·7H_2_O according to the Box-Behnken design (Table 3). The media was sterilized at 121 °C for 15 min. After cooling down the medium, 3% inoculum (*S. cerevisiae*) was added under aseptic conditions. Fermentation experiments were carried out in a shaker incubator at constant temperature and agitation, 150 rpm and 30 °C, respectively, for 48 h. All runs were repeated in triplicate. The ANOVA and regression analysis were conducted to determine the coefficients of the predictive model and significant terms (Minitab Statistical Software, State College, PA, USA). Determination of the optimum medium composition was obtained by the Response Optimizer tool in Minitab Software, and the identified optimum conditions were validated experimentally. 

### 4.5. Analysis

#### 4.5.1. Ethanol and Glucose

The ethanol and glucose concentrations of the fermentation samples were determined using a Waters’ high pressure liquid chromatography (HPLC) equipped with a refractive index detector (Waters, Milford, MA, USA). Bio-Rad Aminex HPX-87H column (300 mm × 7.8 mm; Bio-Rad, Richmond, CA, USA) was used as the HPLC column with 0.8 mL/min of 0.012 N sulfuric acid as mobile phase. The detector and column temperatures were constant at 35 and 65 °C, respectively. Ethanol and glucose concentrations were quantified on the basis of peak area and retention time of the ethanol and glucose standards that were prepared with 200 proof ethanol and D-glucose monomer (1, 5, 10, 20, 30 g/L). Prior to the HPLC analysis, samples were centrifuged at 4 °C for 20 min at 5200× *g* in order to separate the cells and potato particles. The supernatant were filtered with 0.2 μm nylon syringe filters (VWR, Radnor, PA, USA) and injected to HPLC.

#### 4.5.2. Microbial Cell Population 

Cell population was determined with spiral plating method by using a spiral auto-plater (Model 4000, Spiral Biotech, Norwood, MA, USA) and Q-count software (Version 2.1; Spiral Biotech, Norwood, MA, USA). Serial dilution of the samples were carried out with 0.1% sterile peptone water and spirally plated on potato dextrose agar (Difco, Sparks, MD, USA). Plates were incubated at 30 °C for 24 h. Enumeration of the grown colonies were performed by Q-count software (Version 2.1). Results were reported as Log_10_ CFU/mL.

#### 4.5.3. Dry Weight Analysis 

The dry weight of industrial potato waste was determined. Samples were weighed and dried for 48 h at 105 °C in an oven, until constant weight was achieved within 48 h.

## 5. Conclusions

This study presented medium component screening and the effects of medium components on ethanol production and the use of response surface methodology, Box-Behnken design in particular, to determine ethanol production from industrial waste potato mash for various media compositions. Yeast extract, malt extract, and MgSO_4_·7H_2_O were identified as the variables that had statistically significant positive effects on ethanol production by *S. cerevisiae*. Optimal medium composition was determined as 40.4 g/L industrial in waste potato mash hydrolysate, 50 g/L malt extract, and 4.84 g/L MgSO_4_·7H_2_O, and resulted in 24.6 g/L ethanol concentration at 30 °C and 48 h of fermentation. The results imply that the wastes of potato industry can serve as a inexpensive feedstock for ethanol industry. 
